# Glioblastoma Multiforme Occurring After 20 Years at the Resection Site of Cerebral Arteriovenous Malformation: A Case Report and Literature Review

**DOI:** 10.7759/cureus.97919

**Published:** 2025-11-27

**Authors:** Eric O Sarpong, Vitaly Sokotukhin, Hannes Egermann, Adolf Mueller, Landry M Konan

**Affiliations:** 1 Neurological Surgery, Barmherzige Brueder Hospital, Regensburg, DEU; 2 Anatomy and Neurosurgery, Nicklaus Children’s Hospital, Miami, USA

**Keywords:** angiogenic factors, arteriovenous fistula (avf), arteriovenous malformation (avm), cerebral avm, glioblastoma (gbm), glioma

## Abstract

Glioblastoma (GBM) arising at the site of a previously resected arteriovenous malformation (AVM) is exceedingly rare, and its pathophysiological significance remains unclear. Here, we present a unique case of GBM diagnosed two decades after AVM resection and review the literature on AVM-associated, high-grade gliomas. A 67-year-old male presented with headaches and right-sided hemiparesis of progressive onset. Brain MRI revealed a 6 × 4.5 cm heterogeneously enhancing lesion with perifocal edema in the left parietal lobe, precisely where an AVM had been surgically removed 20 years earlier, without adjuvant radiotherapy. The patient underwent gross total resection of the lesion. Histopathological and molecular analysis confirmed GBM, IDH-wild type, with MGMT promoter methylation. To date, nine cases have documented histological coexistence of GBM with AVM or arteriovenous fistula, with one case reporting radiological co-occurrence in distinct brain regions. Additionally, isolated reports describe AVM associated with other high-grade gliomas, including anaplastic variants, either concurrently or sequentially. GBM developing at the site of prior AVM resection is unique, raising questions about a potential biological link. This case underscores the importance of long-term surveillance in AVM patients post-surgery. Further investigation through large-scale cohort studies is warranted to elucidate any mechanistic or oncogenic relationship between AVMs and gliomas, particularly GBM.

## Introduction

Glioblastoma (GBM) is the most common and aggressive primary malignant glioma in adults, typically arising de novo, that is, the tumor occurs in normal brain cells [1-3]. Secondary GBM develops in areas of prior cerebral pathology or intervention and is very rare [1,4]. Arteriovenous malformations (AVMs), congenital vascular anomalies involving direct arteriovenous shunting, are treated via microsurgical resection, embolization, or radiosurgery, primarily to prevent hemorrhage and preserve neurological function [5,6].

High-grade gliomas, however, arising within a previously resected AVM cavity, are exceptionally uncommon [7]. Sparse reports link GBM to other benign intracranial conditions, such as post-traumatic gliosis or resected meningiomas, implicating mechanisms such as chronic gliosis, surgical scarring, and radiation-induced mutagenesis [3,6,8,9].

Here, we report a rare case of GBM diagnosed two decades after AVM resection, without prior irradiation or radiosurgery. This presentation complicates radiological differentiation between recurrent vascular pathology and neoplasm. Documenting such cases may illuminate potential associations between prior vascular lesion treatment and delayed gliomagenesis.

## Case presentation

A 67-year-old male presented to the emergency department with progressive dyscoordination of the right upper and lower extremities and a tendency to veer rightward while ambulating. He also reported a pressure-like headache, predominantly in the early morning hours. The neurological examination showed a subtle right hemiparesis; otherwise, the rest of the examination was unremarkable.

**Figure 1:**
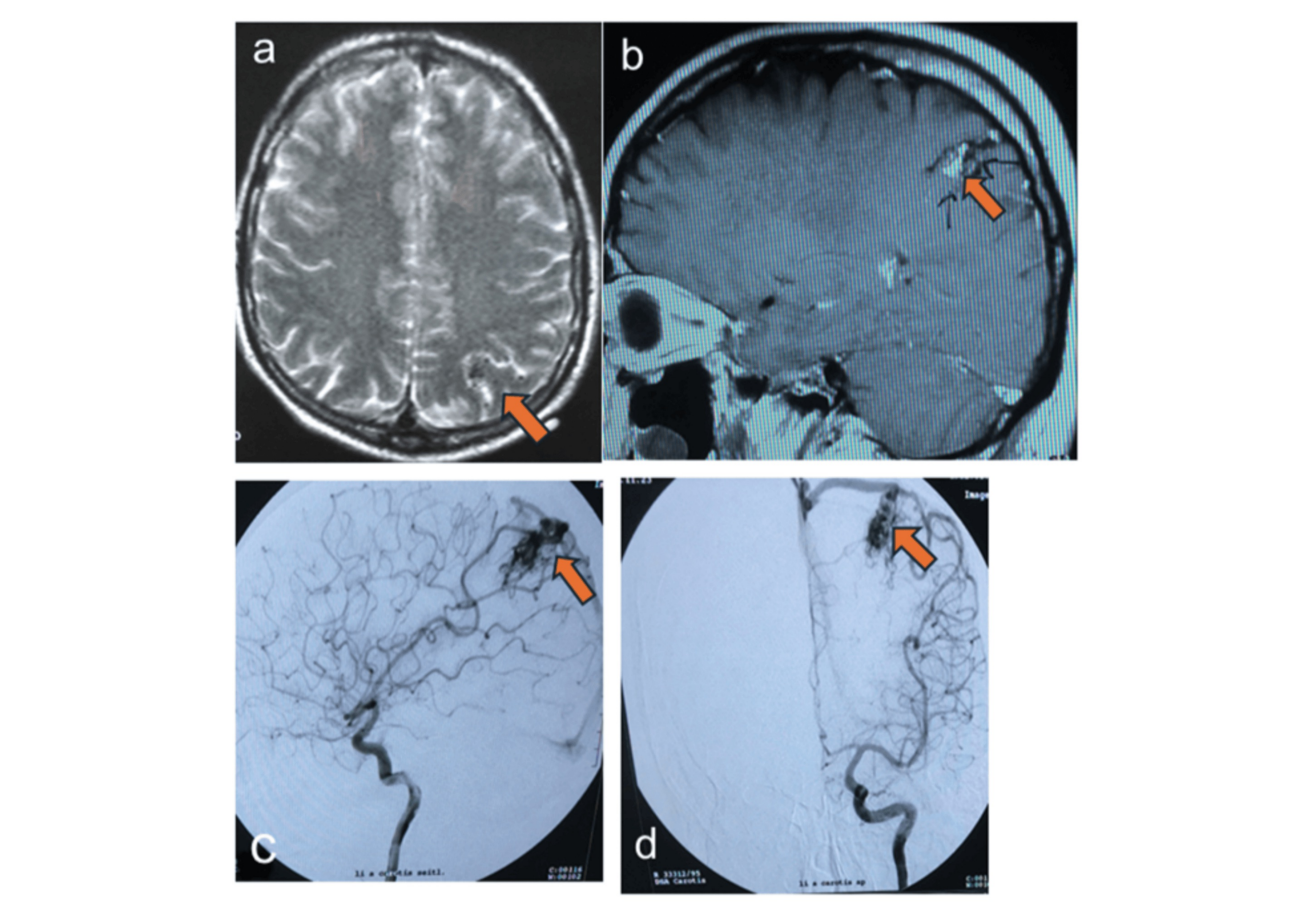
Left parietal arteriovenous malformation. (a) Axial T2-weighted MRI showing subarachnoidal flow voids left parietal (red arrow), indicating vascular structures. There is no parenchymal edema. (b) Post-contrast sagittal T1-weighted MRI showing left parietal contrast-enhancing curvilinear structures analogous to arteriovenous malformation (red arrow). (c, d) Digital subtraction angiography of the left internal carotid artery, lateral (c) and anteroposterior (d) views, showing an arteriovenous malformation with a nidus supplied by the anterior and posterior parietal branches of the inferior ramus of the left middle cerebral artery and the parietal branch of the callosomarginal artery from the left anterior cerebral artery.

His medical history was notable for microsurgical resection of an AVM, Spetzler-Martin grade 2 (1 for eloquence and 1 for size) and supplementary scale grade 5 (compact, unruptured, age more than 40, +3 = supplementary scale grade 5) in the left parietal lobe 20 years prior (Figure 1), complicated by postoperative hemorrhage requiring re-craniotomy. No adjuvant radiotherapy was administered at that time.

**Figure 2:**
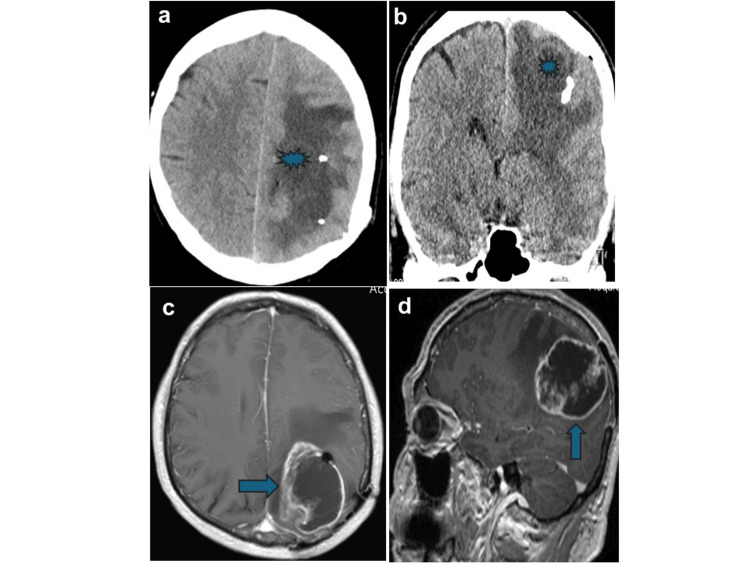
Preoperative cerebral imaging. (a, b) Native axial and coronal CT images showing cerebral edema and a suspected lesion in the left parietal region. (c, d) Axial and sagittal T1-weighted MRI sequences with gadolinium contrast showing a contrast-enhancing lesion with surrounding edema in the left parietal region.

Initial non-contrast head CT revealed a hypodense lesion in the left parietal lobe (Figures 2a, 2b). MRI of the head demonstrated a 6 × 4.5 cm heterogeneously enhancing mass with central necrosis and surrounding vasogenic edema measuring approximately 2.5 cm in width (Figures 2c, 2d). Radiographically, both radiation necrosis and GBM are very similar with contrast enhancement on post-contrast imaging, and most have a mass effect. Radiation necrosis was excluded based on the patient’s absence of prior cranial irradiation. Electroencephalography was unremarkable. Steroid therapy was initiated perioperatively to manage cerebral edema.

**Figure 3:**
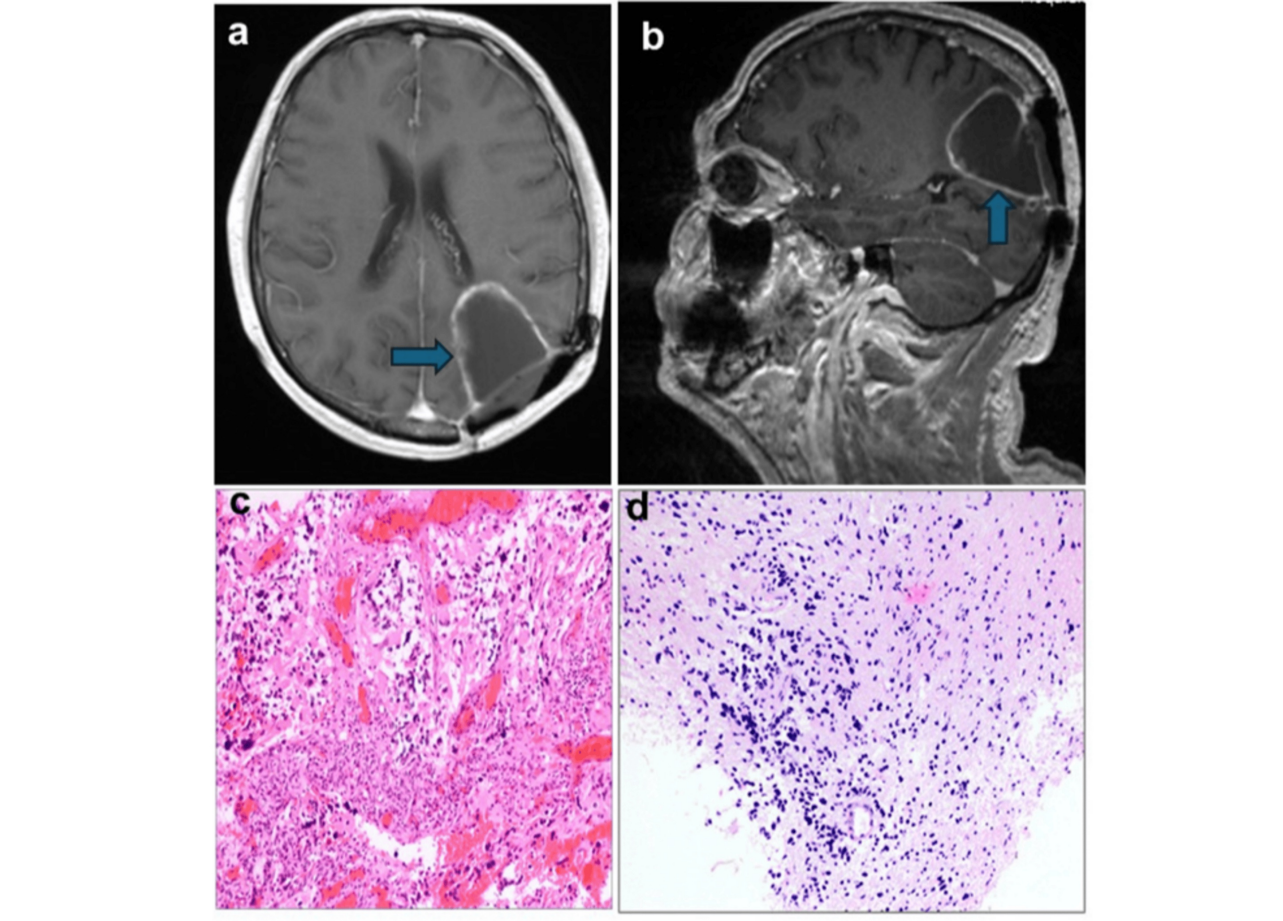
Postoperative MRI and histopathology findings. (a, b) Axial and sagittal T1-weighted magnetization prepared rapid gradient-echo images with gadolinium after tumor resection in the left parietal lobe, showing contrast enhancement around the margins of the resection cavity (arrow). (c) Highly cellular glioblastoma demonstrating nuclear pleomorphism and glomeruloid vascular proliferation. In some areas, a gemistocytic appearance is observed (100×). (d) Overview (100×): diffuse high-grade astrocytic glioma with high cellularity and hyperchromatic nuclei.

**Figure 4:**
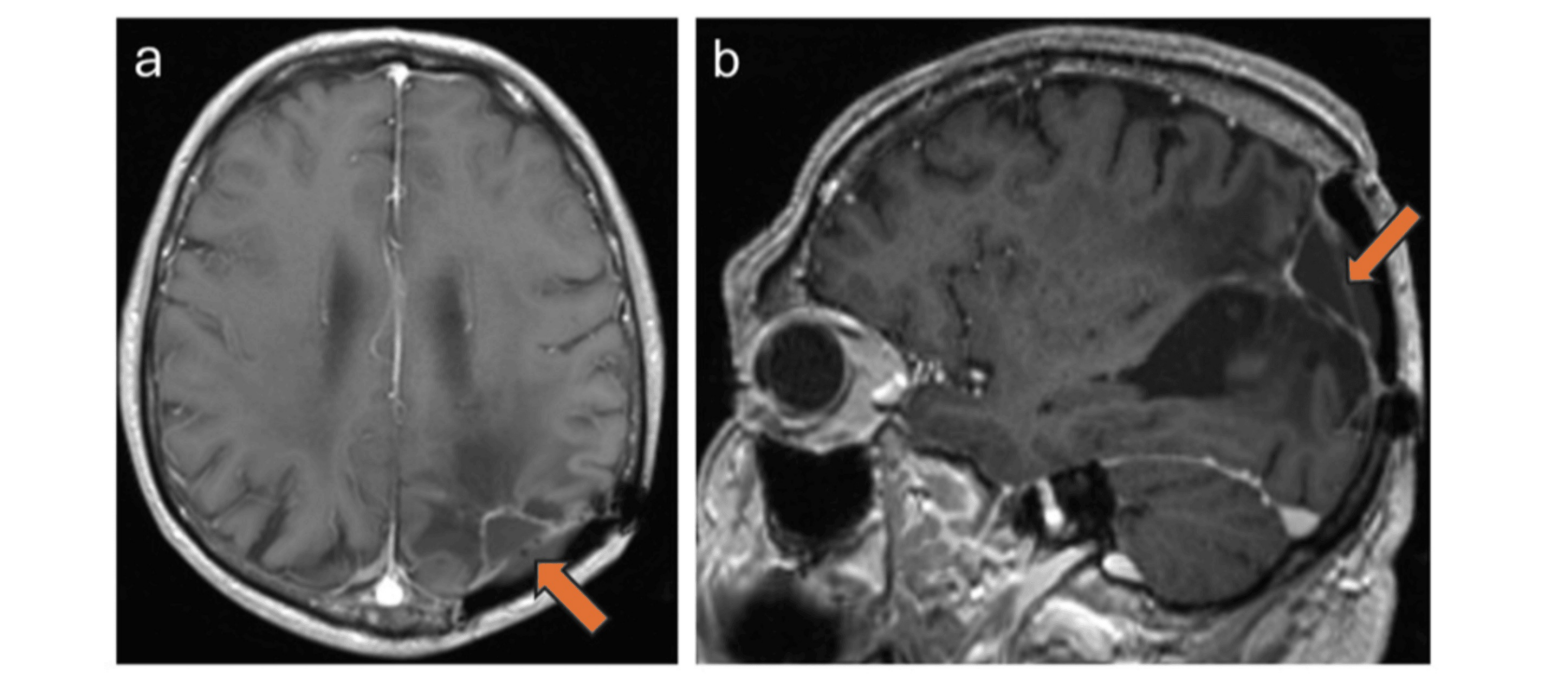
Radiographic imaging five years after surgery and radiation therapy. (a) Axial T1-weighted MR image with contrast and (b) sagittal T1-weighted MPRAGE image showing postoperative and post-radiation changes in the left parietal lobe. Note the contrast enhancement at the margins of the resection cavity (arrow).

The patient was admitted to the neurosurgical service and underwent a re-craniotomy of the left parietal lobe in the concorde position. The approach was a parieto-occipital craniotomy with a curved incision through the scarred region from the previous surgery. The dura mater in this region was scarred. After an ultrasound sonographic examination of the tumor, a microsurgical gross total resection of the lesion using 5-ALA was achieved without intraoperative complications. Histopathological and molecular analysis confirmed glioblastoma multiforme (GBM), IDH-wild type, with MGMT promoter methylation. Postoperative MRI at six months demonstrated contrast enhancement along the margins of the resection cavity (Figure 3). The patient underwent standard concurrent chemoradiotherapy per the Stupp protocol. The patient remains neurologically and radiologically stable at the five-year follow-up (Figure 4).

## Discussion

Coincidences of a cerebral AVM and a GBM are very rare [4,6,8,10,11]. To our knowledge, a total of about 16 cases of association between vascular malformations (AVM, dural arteriovenous fistula (DAVF)) and GBM have been reported (Table 1). Additionally, two cases of anaplastic astrocytoma were reported in association with AVM (see Table 1). The median age of all 18 cases was 55 years (range = 9-72 years). Overall, 44% and 56% of the cases were female and male, respectively, with a male to female ratio of 1,2:1. The occurrence of these lesions can be classified as follows: (1) both entities occurring concomitantly at the same location, referred to as a “collision tumor”; (2) both entities occurring at different locations in the brain; or (3) sequential occurrence of both entities at the same location. In 16 of the 18 cases (89%), thorough histopathological studies revealed that both pathologies were concomitantly present in the tumor (see Table 1).

**Table 1:** A literature review of reported cases of AVMs associated with GBM or anaplastic astrocytoma. AV = arteriovenous; DAVF = dural arteriovenous fistula; AVM = arteriovenous malformation; GBM = glioblastoma multiforme

Reference	Age of the patient	Sex of the patient	Radiological findings		Type of association	Histology of the lesion
			Tumor	Vascular malformation		
Imai et al., 2016 [1]	66	Male	GBM (CT)	AV shunt (angiogram)	Combined	Hypervascular GBM
Boikov et al., 2014 [2]	29	Female	-	AV fistula	Combined	GBM, AV fistula
Tunthanathip, et al., 2020 [3]	20	Male	Hypervascular lesion	AVM	Combined	GBM, AVM
Aucourt et al., 2012 [6]	65	Male	High-grade glioma	AVM	Combined	GBM, AVM
Goodkin et al., 1990 [7]	9	Female	-	AVM	Sequentiall	Anaplastic astrocytoma
Lohkamp et al., 2016 [8]	71	Female	GBM (MRI)	AVM (CT angiography)	Combined	GBM
Hubell et al., 1961 [9]	70	Male	-	AVM	Combined	GBM, AVM
Cemil et al., 2009 [10]	58	Male	-	AVM	Combined	Hypervascular
Zucarrello et al., 1979 [12]	50	Male	Temporal mass lesion	AV fistula	Combined	GBM, AVM
Licata et al., 1986 [13]	60	Female	GBM left temporal	AVM left occipital	Separate	GBM
Harris et al., 2000 [14]	57	Male	-	AVM	Combined	Anaplastic astrocytoma
Ziyal et al., 2004 [15]	58	Male	High-grade glioma	-	Combined	AVM, high-grade glioma
Gmeiner et al., 2013 [16]	72	Female	High-grade glioma	-	Combined	AVM, GBM
Khanna et al., 2013 [17]	53	Male	-	AVM with ICB	Combined	GBM, AVM
Linsenmann et al., 2015 [18]	35	Male	GBM	AVM	Combined	Primary spinal GBM, 2° cranial GBM
Yuan et al., 2016 [19]	23	Female	High-grade glioma	DAVF	Combined	GBM, DAVF
Brock et al., 2017 [20]	46	Female	GBM	-	Combined	AVM, GBM
Li CR et al., 2023 [21]	44	Female	GBM, AVM	AVM, aneurysm	Combined	-
Present case	67	Male	-	AVM	subsequent	GBM

In most cases, however, the brain tumor was preoperatively considered solely as an AVM or a GBM [7,8,16,17,19]. The other histological component was first diagnosed intraoperatively or postoperatively [10,19]. In a case reported by Licata et al., both tumor types were concomitantly present in different locations of the brain [13]. In this study [13], they reported another case of a 44-year-old man who initially had a rolandic angioma and developed a low-grade glioma at the same location 30 months later.

Furthermore, AVM and GBM can present sequentially at the same location, and either of the lesions can precede the other [7,13,14,22]. In this review, one patient with a sequential occurrence of GBM and AVM (5.5%) was reported. Goodkin et al. also described a case of sequential occurrence involving an AVM and an anaplastic glioma, in which resection of the AVM preceded the diagnosis of the glioma at the same site [7]. Beyond the AVM entity, the sequential occurrence of other vascular malformations and glioma has also been reported in the literature [14,22]. A recently reported case had three lesions (GBM, aneurysm, and AVM) in one entity [21]. This case study examines the phenomenon of the sequential occurrence of GBM and AVM. To date, no case has been reported of a sequential AVM and GBM with a large time interval of about 20 years. In the pathohistological examination of both entities, a coexistence of AVM and GBM was excluded. The histopathological features of the GBM are shown in Figures 3c, 3d.

The pathogenesis of GBM is due to a combination of genetic changes, such as loss of tumor suppressor genes and modified angiogenetic pathways [14,23,24]. In vascular malformations such as AVMs, DAVF, and cerebral cavernous malformations (CCMs), neovascularization and angiogenesis are major mechanisms that are implicated in the pathogenesis [14,25].

The etiologies of the existence of vascular malformation and gliomas are multiple [7]. These include accidents, genetic changes, and secondary or neoplastic glial changes [7]. It cannot be ruled out that the sequential occurrence of the AVM and GBM in the same location is coincidental; however, due to some similar pathogenetic mechanisms of both lesions, clues are obtained as to how one lesion can contribute to the formation of the other [13,14,19]. The existence of one of the two lesions is a risk factor for the occurrence of the other [3]. AVM-related hemorrhages may cause glial cell differentiation and reactivation [12,17]. The stress on the glial tissues due to AVM is a plausible hypothesis for the sequential occurrence of AVM and GBM [16,20,26].

Finally, there are cytokines and other angiogenic factors implicated in GBM and vascular lesions such as CCMs and AVMs [24,27-29]. AVM and CCMs release and respond to cytokines, such as vascular endothelial growth factors (VEGFs), fibroblast growth factor, angiogenin, and tumor necrosis factor-alpha, that promote neoplasia [14,27]. VEGF can promote angiogenesis in GBM tumors and promote the formation and growth of AVMs [14,19,27]. Therefore, it may be plausible to think that similar molecular interactions, such as VEGF secretions, can also promote the formation of an AVM within tumors [16,19,27-29].

## Conclusions

This case study explores the rare occurrence of GBM developing two decades after the resection of an AVM in a 67-year-old male. The emergence of high-grade gliomas within a previously resected AVM cavity is exceptionally uncommon. In this case, the patient presented with progressive dyscoordination and headaches, and MRI revealed a lesion in the left parietal lobe, where the AVM had been removed 20 years earlier. Histopathological analysis confirmed GBM, IDH-wild type, with MGMT promoter methylation, and no residual AVM was found. The patient underwent standard chemoradiotherapy and remained stable at a five-year follow-up. Coincidences of cerebral AVM and GBM are very rare, with about 16 cases reported in the literature. These lesions can occur concomitantly, sequentially, or in different locations. The stress on glial tissues due to AVM and cytokines such as VEGF may contribute to tumor formation. Despite theoretical aggressiveness, the patient’s GBM was effectively managed, highlighting the importance of individual factors and timely intervention. Further research is needed to understand the relationship between AVMs and gliomas, which could improve prognosis and treatment strategies.
